# Osteo-Porosis

**Published:** 1886-04

**Authors:** H. F. James

**Affiliations:** St. Louis, Mo.


### Art. VIII.—OSTEO-POROSIS.

BY H. F. JAMES, V.S., ST. LOUIS, MO.

Since Varnell's admirable account of the disease known as Osteo-Porosis, very little has been written on the subject This is a matter of surprise,as a large number of regulars have been practising for years in those sections of the Mississippi Valley where the disease appears most frequently, and with the exception of an able paper by Dr. Adam Harthill, of Louisville, Ky., read before the student association of the Ontario Veterinary College in February, 1884, I am not aware that much has been recorded concerning this malady. Young horses are most frequently attacked, but neither age nor sex affords any protection. It is presumed that the phosphate of lime of the system is discharged in the urine, but analyses have as yet been too imperfect in detail and limited in number to enable me to speak with certainty on that point. The first stage simulates rheumatism so strongly that diagnosis is not only difficult but impossible, unless we take the position held by many that we cannot have rheumatism without swelling of the affected parts.

The first suspicion of osteo-porosis is usually excited by a tucking-up of the flanks and a general look of unthriftiness, the signs of mal-assimilation being rapidly developed. A short, stiff gait is shown, perhaps a little lameness in one or more of the limbs which may shift or entirely disappear, and the disease be in abeyance for months. The idea that the bones of the head and shoulders are first affected is an erroneous one. I have seen well-marked cases in which not the slightest swelling or tenderness of these parts could be detected; but as a general thing it shows itself by a swelling of the bones of the face or a thickening of the ramus of the lower jaw sooner or later. There is rigidity of the loins, and the resemblance to a rheumatic attack is very close. But the symptoms do not yield to anti-rheumatic medication; marasmus increases, perhaps a little puffy swelling occurs in either a fore or a hind fetlock, and all of a sudden when making a hasty step, the perforans is detached from the os pedis, carrying small fragments of bone away with it, the sesamoids fracture, the suspensories tearing away small pieces of bones, or any part of the osseous system may give way from an apparently trivial cause. I have seen a horse fracture both femurs starting a heavy load, another one going only a little stiff fall in a spring wagon and fracture the bodies of four dorsal vertebrae; in these cases of course the diseased process was of some standing. A post-mortem usually shows the slate-colored appearance of certain of the articulations. A peculiar feature of osteo-porosis is the rapidity with which the disease may be developed in an apparently healthy animal. The exciting cause may be a wound of any kind, most frequently a nail in the foot. The puncture is well pared out, soaked in hot water and poulticed; everything connected with the local treatment is done thoroughly and yet the patient does not bear much weight upon the limb. You again pare the part thoroughly and search for deep seated suppuration, find nothing and perhaps conclude that subacute periostitis of the pedes has set in, more hot poultices give no relief, you come in some morning and your patient standing pretty well on the foot, but now propped up by both fore legs in an exaggerated founder attitude. There is no heat of the feet, no sensitiveness to tapping with the hammer, and you quickly satisfy yourself that there is no congestion of the laminæ. The pedal bones are becoming affected, osteo-porosis has been brought on in some occult manner by the irritation of the puncture; the predisposition to the disease being already in the system. Marasmus increases, the animal is continually stretching backwards in a vain attempt to obtain relief from the excruciating agony it suffers, the appetite is poor, sometimes the poor beast will starve to death if the bones of the head are badly affected, decubitus is frequent, finally a fracture occurs, ligaments and tendons are torn away from the more diseased bones and the usual termination is reached. Implication of the pedal bones is very common where the taint is in the economy, and the exaggerated founder attitude which I have before referred to, and which is seen more often in the front limbs is very characteristic. These cases used to puzzle me somewhat when I first came west, but I soon recognized my enemy. I have seen it develop very quickly after a bad attack of acute indigestion. Situation does not seem to have much to do with its appearance. I had 18 or 20 cases not long since in a stable with a dirt floor. The drainage was poor, but I do not think that has much to do with it; the food is probably responsible. It is becoming rarer every year as the land is being better cultivated, and it is not dreaded here as it was formerly. The possible existence of osteo-porosis should always be allowed for in diagnosing apparently rheumatic troubles in horses coming from the Mississippi valley, the absence of swelling of the bones of the head is by no means to be regarded as positive evidence of freedom from the taint. Treatment is absolutely useless, neither purgatives, tonics, nor anything else will restore proper nutrition, and death from marasmus or disability from the giving way of diseased structures is the inevitable result.

